# Supplementary figures and images for: Selection and Validation of Reference Genes for Gene Expression Studies in an Equine Adipose-Derived Mesenchymal Stem Cell Differentiation Model by Proteome Analysis and Reverse-Transcriptase Quantitative Real-Time PCR
Source: Genes (Basel). 2023 Mar 8;14(3):673. doi: 10.3390/genes14030673 (PMC10048155; doi:10.3390/genes14030673)

Determination of the optimal number of reference targets

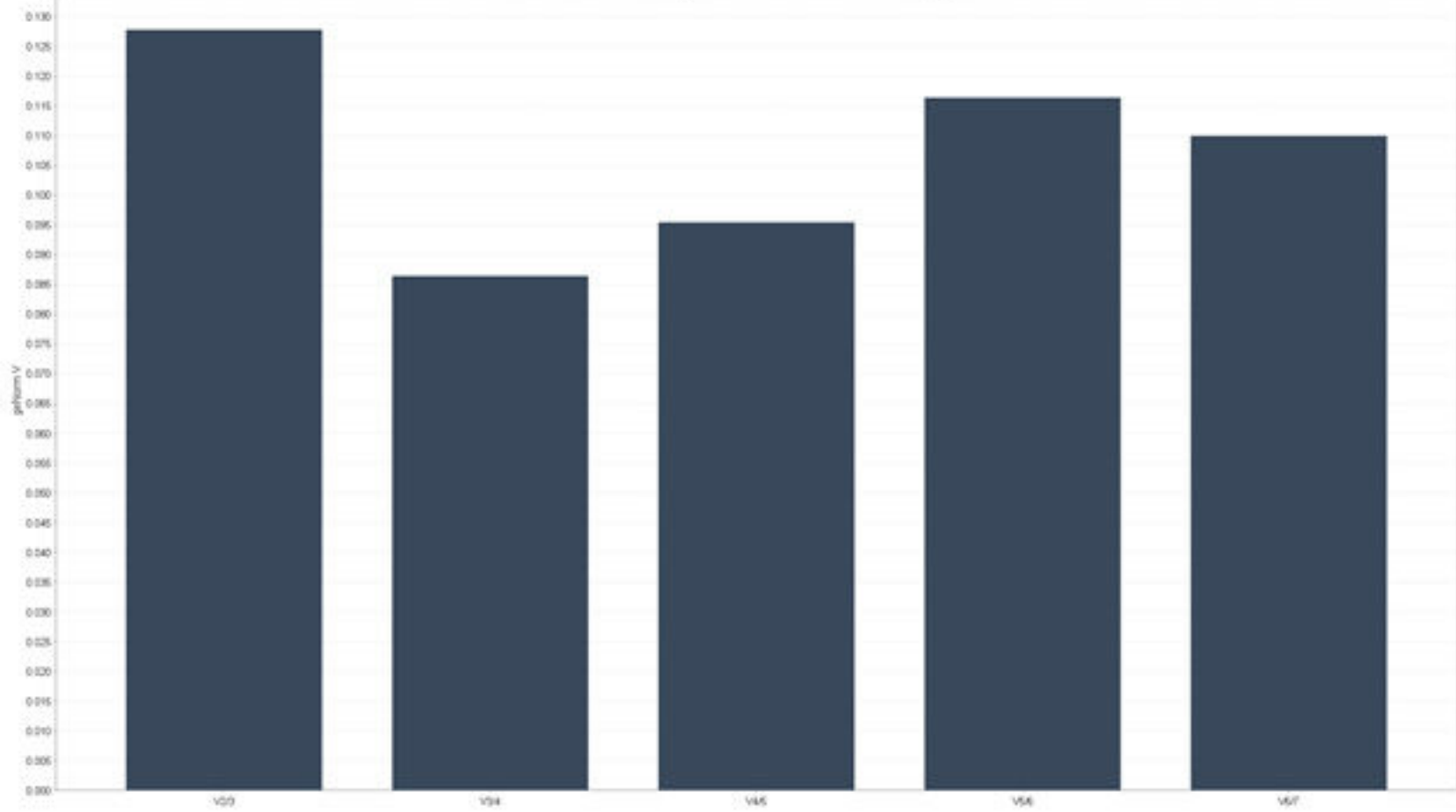

Supplement: Supplementary file 1 [file genes-14-00673-s001.zip › genes-2252351-supplementary/SF1.pdf]
